# A universal Bayesian inference framework for complicated creep constitutive equations

**DOI:** 10.1038/s41598-020-65945-7

**Published:** 2020-06-26

**Authors:** Yoh-ichi Mototake, Hitoshi Izuno, Kenji Nagata, Masahiko Demura, Masato Okada

**Affiliations:** 1 The Institute of Statistical Mathematics, Tachikawa, Tokyo 190-8562 Japan; 2 Research and Services Division of Materials Data and Integrated System, National Institute for Materials Science, Namiki 1-1, Tsukuba, Ibaraki 305-0044 Japan; 3 Graduate School of Frontier Sciences, University of Tokyo, Kashiwa, Chiba 277-8561 Japan

**Keywords:** Materials science, Structural materials, Metals and alloys

## Abstract

Evaluating the creep deformation process of heat-resistant steels is important for improving the energy efficiency of power plants by increasing the operating temperature. There is an analysis framework that estimates the rupture time of this process by regressing the strain–time relationship of the creep process using a regression model called the creep constitutive equation. Because many creep constitutive equations have been proposed, it is important to construct a framework to determine which one is best for the creep processes of different steel types at various temperatures and stresses. A Bayesian model selection framework is one of the best frameworks for evaluating the constitutive equations. In previous studies, approximate-expression methods such as the Laplace approximation were used to develop the Bayesian model selection frameworks for creep. Such frameworks are not applicable to creep constitutive equations or data that violate the assumption of the approximation. In this study, we propose a universal Bayesian model selection framework for creep that is applicable to the evaluation of various types of creep constitutive equations. Using the replica exchange Monte Carlo method, we develop a Bayesian model selection framework for creep without an approximate-expression method. To assess the effectiveness of the proposed framework, we applied it to the evaluation of a creep constitutive equation called the Kimura model, which is difficult to evaluate by existing frameworks. Through a model evaluation using the creep measurement data of Grade 91 steel, we confirmed that our proposed framework gives a more reasonable evaluation of the Kimura model than existing frameworks. Investigating the posterior distribution obtained by the proposed framework, we also found a model candidate that could improve the Kimura model.

## Introduction

Evaluating the creep deformation process of heat-resistant steels is important for improving the energy efficiency of power plants by increasing the operating temperature^1,2^. One of the most effective frameworks for estimating this process is the creep constitutive equation approach. On the basis of the regression result of the strain–time relationship using a regression model called the creep constitutive equation^1–9^, the approach predicts the transition of a creep deformation process obeying the alternation of temperature and stress. It has been reported that the creep constitutive equation approach can predict the quantities required to evaluate creep phenomena, such as rupture time, with high accuracy. Because many creep constitutive equations have been proposed, it is important to construct a framework to determine which one is best for the creep processes of different steel types at various temperatures and stresses. Hereinafter, this problem is called creep model selection for simplicity.

Bayesian model selection is one of the best frameworks for evaluating such creep constitutive equations because it can select the model (=equation) that minimizes the fitting error while avoiding model complexity^10^ (see Appendix A). Evaluating the model on the basis of only the fitting error tends to result in the selection of a more complex model, which leads to overfitting^10^. Overfitting the creep measurement data under specific temperature and stress conditions would make it difficult to model the transition of a creep deformation proccess obeying the alternation of temperature and stress. Bayesian model selection has another advantage, that is, the model parameters are estimated as probabilistic variables rather than point values. The maximum-likelihood methods, such as the least-squares method, estimate model parameters as point values. The probabilistic treatment of parameters introduces both prior and posterior distributions of parameters in the model selection framework. The prior distributions model the prior knowledge of parameters. For example, we can add knowledge, such as the range of a parameter, into the model. The posterior distributions are the estimation results of parameters based on measurement data. From the posterior distributions, we can obtain the estimation reliability of the parameters or obtain the properties of the model, such as the correlations among the parameters. These cannot be obtained by a maximum-likelihood method. Such knowledge helps to design a measurement plan or comprehend the model^11,12^.

In a previous study^13^, we applied a Bayesian model selection framework for the evaluation of creep constitutive equations constructed from linear sums of basis functions. We computed two simple creep constitutive equations with and without a steady-state term, and found that the equation with a steady-state term was selected over a wide temperature and stress range for Grade 91 (Gr.91) steel^14^, a high-Cr, ferritic, heat-resistant steel. For a more accurate prediction of creep phenomena, it is necessary to apply a more sophisticated constitutive equation. There are several constitutive equations that have been proposed so far. Recently, Kimura *et al*. have proposed a novel constitutive equation^1,2^ and showed that it reproduces the experimental creep curve very well. The creep constitutive equation is formulated as

1 $$\varepsilon ({t}_{i};{{\boldsymbol{\theta }}}_{{\rm{kimura}}})={\varepsilon }_{0}+{a}_{1}{t}^{{b}_{1}}+{a}_{2}{t}^{{b}_{2}}+{c}_{1}\,\exp ({d}_{1}t)+{c}_{2}\,\exp ({d}_{2}t),$$

where $\varepsilon$ is the strain, $t$ is the time, and ${{\boldsymbol{\theta }}}_{{\rm{kimura}}}$ is the parameter set $\{{\varepsilon }_{0},{a}_{1},{a}_{2},{b}_{1},{b}_{2},{c}_{1},{c}_{2},{d}_{1},{d}_{2}\}$ of the creep constitutive equation. We refer to this creep constitutive equation as the Kimura model. Thus, the Kimura model consists of a linear sum of the four basis functions that depend on time $t$. This is one of the creep constitutive equations with the largest number of parameters among the equations that have been proposed so far^15,16^. Our previous framework is not applicable to such sophisticated or complicated creep constitutive equations. This is because the parameters of the exponential part of each basis function are not treated as probabilistic variables in the previous framework: the parameters are optimized by a grid search and point estimation based on the empirical Bayes method^10^. If the number of grids per axis is $G$ and the number of parameters is $d$, the computational cost to execute Bayesian model selection is ${G}^{d}$. Therefore, it is not realistic to apply the previous framework to a model with many basis functions, such as the Kimura model. Treating the parameters of a basis function as deterministic parameters is an approximation-expression method in which the posterior distribution of the parameters is a delta function, which is called as the empirical Bayes approximation. If the parameters of the basis function are not well-determined, the empirical Bayes approximation generates a bias in the model selection result. The exchange symmetry of parameter pairs, such as ${a}_{1}$ - ${a}_{2}$, ${b}_{1}$ - ${b}_{2}$, ${c}_{1}$ - ${c}_{2}$, and ${d}_{1}$ - ${d}_{2}$ in the Kimura model, is an example of parameters that are not well-determined. Keitel *et al*. also applied a Bayesian model selection framework for the model selection of creep constitutive equations of concrete^17^, where they approximated the posterior distribution of parameters as a Gaussian. Such the approximate-expression method is called the Laplace approximation. The Laplace approximation also leads to a false conclusion if the model parameters are not well-determined^10^. To evaluate the complex creep constitutive equations, it is necessary to perform Bayesian creep model selection without an approximate-expression method. There is another difficulty in Bayesian creep model selection. In the measurement of the creep deformation process in steel, it is difficult to estimate the measurement noise intensity correctly; instead, a noise range is given. In creep model selection, it is important to set the noise intensity correctly, because the noise intensity is related to a criterion that determines whether the signal is to be regressed or considered as noise. To set the noise intensity as a range, it is necessary to set the noise intensity as a probabilistic variable and set its range as a prior distribution. However, in the existing creep model selection frameworks, it is difficult to treat the noise intensity as a probabilistic value. Thus, there is no Bayesian creep model selection framework that can be used to evaluate all types of creep constitutive equations correctly.

In this study, using the replica exchange Monte Carlo method^18^, we propose a universal Bayesian creep model selection framework that can be applied to various types of creep constitutive equations without the approximate-expression method or the ability to set the range of the measurement noise intensity as a prior distribution. By applying the proposed framework to the evaluation of the Kimura model using the creep measurement data of Gr.91 steel^14^, we confirmed that our proposed framework gives a more reasonable evaluation of the Kimura model than existing frameworks. From the accurate posterior distribution obtained by the proposed framework, we also found a way to simplify the Kimura model without losing the model likelihood.

## Methods

**Figure 1:**
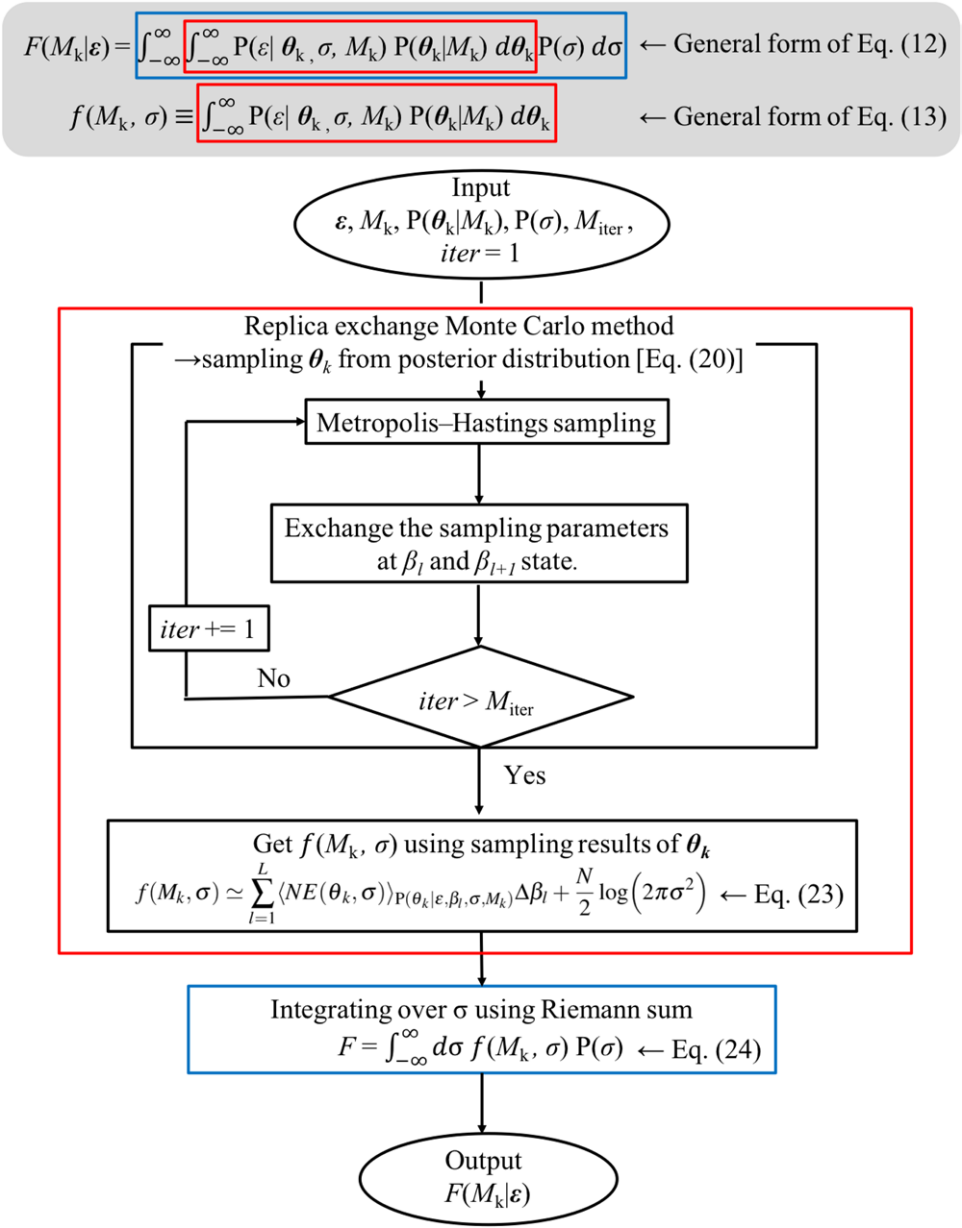
Flowchart of proposed framework.

In the proposed framework, the criterion to achieve a Bayesian creep model selection is obtained by numerical integration. The numerical integration consists of a sequential run of numerical integration using the REMC method and the Riemann sum. The flowchart of the framework is shown in Fig. 1.

### Bayesian creep model selection

We evaluate $K$ creep constitutive equations ${M}_{k}$ in terms of their ability to represent the creep deformation data ${\bf{D}}=\{{\bf{t}},{\boldsymbol{\varepsilon }}\}=\{({t}_{1},{t}_{2},\ldots {t}_{N}),({\varepsilon }_{1},{\varepsilon }_{2},\ldots {\varepsilon }_{N})\}$, where $\varepsilon$ is the strain and $t$ is the time. The likelihood of a given creep constitutive equation ${M}_{k}(k=1,\ldots K)$ for the creep deformation data *D* is

2 $${\rm{P}}({M}_{k}|{\bf{D}})=\frac{{\rm{P}}({\bf{D}}|{M}_{k}){\rm{P}}({M}_{k})}{{\rm{P}}({\bf{D}})}\propto {\rm{P}}({\bf{D}}|{M}_{k}){\rm{P}}({M}_{k}),$$

where ${\rm{P}}({\bf{D}})$ is a normalization constant. In this study, we assume that there is no prior knowledge about the likelihood of the model. Thus, we set the prior probability ${\rm{P}}({M}_{k})$ as a uniform distribution; in this study, it is equal to $\frac{1}{K}$. We also assume that **t** in the dataset ${\bf{D}}=\{{\bf{t}},\varepsilon \}$ is given deterministically, that is, non-probabilistically. Then, on the basis of Bayes’ theorem, the likelihood of the model is transformed as

3 $${\rm{P}}({M}_{k}|{\bf{D}})={\rm{P}}({M}_{k}|{\boldsymbol{\varepsilon }},{\bf{t}})={\rm{P}}({M}_{k}|{\boldsymbol{\varepsilon }})\propto {\rm{P}}({\boldsymbol{\varepsilon }}|{M}_{k})$$

4 $$\,={\int }_{-\infty }^{\infty }\,{\int }_{-\infty }^{\infty }\,{\rm{P}}({\boldsymbol{\varepsilon }}|{{\boldsymbol{\theta }}}_{k},\sigma ,{M}_{k}){\rm{P}}({{\boldsymbol{\theta }}}_{k}|{M}_{k}){\rm{P}}(\sigma )d{{\boldsymbol{\theta }}}_{k}d\sigma ,$$

where ${{\boldsymbol{\theta }}}_{k}$ is the parameter set of the creep constitutive equation ${M}_{k}$ and $\sigma$ is the noise intensity.

The conditional probability ${\rm{P}}({\boldsymbol{\varepsilon }}|{{\boldsymbol{\theta }}}_{k},\sigma ,{M}_{k})$ of Eq. (4) is a stochastic generative model of the creep constitutive equation ${M}_{k}$. When the measurement noise of the creep deformation data is given as an independent and identically distributed Gaussian with average 0 and standard deviation $\sigma$, the conditional probability can be expressed as

5 $${\rm{P}}({\boldsymbol{\varepsilon }}|{{\boldsymbol{\theta }}}_{{\bf{k}}},\sigma ,{M}_{k})=\mathop{\prod }\limits_{i=1}^{N}\,{\rm{P}}({\boldsymbol{\varepsilon }}|{{\boldsymbol{\theta }}}_{k},\sigma ,{M}_{k})\,$$

6 $$\,={\left(\frac{1}{2\pi {\sigma }^{2}}\right)}^{N/2}\,\mathop{\prod }\limits_{i=1}^{N}\,\exp \left[-\frac{1}{2{\sigma }^{2}}{({{\boldsymbol{\varepsilon }}}_{i}-{{\boldsymbol{\varepsilon }}}_{k}({t}_{i};{{\boldsymbol{\theta }}}_{k}))}^{2}\right]$$

7 $$\,={\left(\frac{1}{2\pi {\sigma }^{2}}\right)}^{N/2}\exp \left\{\,-\,\mathop{\sum }\limits_{i=1}^{N}\,\left[\frac{1}{2{\sigma }^{2}}{({{\boldsymbol{\varepsilon }}}_{i}-{{\boldsymbol{\varepsilon }}}_{k}({t}_{i};{{\boldsymbol{\theta }}}_{k}))}^{2}\right]\right\},$$

where ${\varepsilon }_{k}({t}_{i};{{\boldsymbol{\theta }}}_{k})$ is the regression function of the creep constitutive equation ${M}_{k}$ as described in the introduction. The probabilities ${\rm{P}}({{\boldsymbol{\theta }}}_{k}|{M}_{k})$ and ${\rm{P}}(\sigma )$ in Eq. (4) respectively simulate the prior knowledge about the model parameters ${{\boldsymbol{\theta }}}_{k}$ and the noise intensity $\sigma$ as probability distributions. By substituting Eq. (7) into Eq. (4), we obtain

8 $$\begin{array}{rcl}{\rm{P}}({\boldsymbol{\varepsilon }}|{M}_{k}) & = & {\int }_{-\infty }^{\infty }\,d\sigma {\left(\frac{1}{2\pi {\sigma }^{2}}\right)}^{N/2}\,{\int }_{-\infty }^{\infty }\,d{{\boldsymbol{\theta }}}_{k}\\  &  & \exp \left\{-\frac{1}{2{\sigma }^{2}}\,\mathop{\sum }\limits_{i=1}^{N}\,{({{\boldsymbol{\varepsilon }}}_{i}-{{\boldsymbol{\varepsilon }}}_{k}({t}_{i};{{\boldsymbol{\theta }}}_{k}))}^{2}\right\}{\rm{P}}({{\boldsymbol{\theta }}}_{k}|{M}_{k}){\rm{P}}(\sigma )\end{array}\,$$

9 $$\,={\int }_{-\infty }^{\infty }\,d\sigma {\left(\frac{1}{2\pi {\sigma }^{2}}\right)}^{N/2}\,{\int }_{-\infty }^{\infty }\,d{{\boldsymbol{\theta }}}_{k}\,\exp [\,-\,NE({{\boldsymbol{\theta }}}_{k},\sigma )]{\rm{P}}({{\boldsymbol{\theta }}}_{k}|{M}_{k}){\rm{P}}(\sigma )$$

10 $$={\int }_{-\infty }^{\infty }\,d\sigma \,\exp \,[\,-\,f({M}_{k},\sigma )]P(\sigma ),\,$$

where

11 $$E({{\boldsymbol{\theta }}}_{k},\sigma )=\frac{1}{2N{\sigma }^{2}}\,\mathop{\sum }\limits_{i=1}^{N}\,{({\varepsilon }_{i}-{\varepsilon }_{k}({t}_{i};{{\boldsymbol{\theta }}}_{k}))}^{2},$$

12 $$f({M}_{k},\sigma )=-\,\log \left\{{\left(\frac{1}{2\pi {\sigma }^{2}}\right)}^{N/2}\,{\int }_{-\infty }^{\infty }\,d{{\boldsymbol{\theta }}}_{k}\,\exp [-NE({{\boldsymbol{\theta }}}_{k},\sigma )]{\rm{P}}({{\boldsymbol{\theta }}}_{k}|{M}_{k})\right\}.$$

The probability ${\rm{P}}({\boldsymbol{\varepsilon }}|{M}_{k})$ is often referred to as the marginal likelihood and is proportional to the likelihood of the recognition model ${M}_{k}$. The negative log-likelihood

13 $$F({M}_{k})=-\,\log \,{\rm{P}}({\boldsymbol{\varepsilon }}|{M}_{k})$$

is often referred to as the Bayesian free energy. In this way, $F({M}_{k})$ is proportional to the negative log-likelihood of the recognition model ${M}_{k}$. Therefore, the creep constitutive equation ${M}_{k}$ with the smallest $F({M}_{k})$ value represents the best model.

### Replica exchange Monte Carlo sampling method

To obtain the value of $F({M}_{k})$, we need to execute the integration in Eq. (4). However, it is difficult to analytically execute the integration owing to the complicated relationship between ${\varepsilon }_{k}({t}_{i};{{\boldsymbol{\theta }}}_{k})$ and ${{\boldsymbol{\theta }}}_{k}$. We overcame this difficulty by numerical integration. The numerical integration was performed in two steps. The first step is integration with respect to the model parameter set ${{\boldsymbol{\theta }}}_{k}$ to obtain the value of $f({M}_{k},\sigma )$ with $\sigma$ [Eq. (12)], and the second step is, by using the obtained $f({M}_{k},\sigma )$ value, integration with respect to the noise intensity $\sigma$ [Eq. (10)] to obtain $F({M}_{k})$. Since step 2 is a one-variable integral with respect to $\sigma$ [Eq. (10)], the numerical integration can be performed as a Riemann sum. On the other hand, since the ${{\boldsymbol{\theta }}}_{k}$ integral is high-dimensional, we carried out the integration by the sampling method. In this section, we explain how to calculate $f({M}_{k},\sigma )$ with a given noise intensity $\sigma$ by the sampling method.

Markov chain Monte Carlo (MCMC) methods^19^ are efficient sampling methods for estimating the expectation value of a probability distribution in a high-dimensional space. $f({M}_{k},\sigma )$ is given using an auxiliary variable $\beta$;

14 $$f({M}_{k},\sigma )=-\,\log \,{\int }_{-\infty }^{\infty }\,\exp [-NE({{\boldsymbol{\theta }}}_{k},\sigma )]{\rm{P}}({{\boldsymbol{\theta }}}_{k}|{M}_{k})d{{\boldsymbol{\theta }}}_{k}+\frac{N}{2}\,\log (2\pi {\sigma }^{2})\,$$

15 $$\,={\int }_{0}^{1}\,\frac{\partial }{\partial \beta }\{-\log [{\int }_{-\infty }^{\infty }\,\exp (\,-\,\beta NE({{\boldsymbol{\theta }}}_{k},\sigma )){\rm{P}}({{\boldsymbol{\theta }}}_{k}|{M}_{k})d{{\boldsymbol{\theta }}}_{k}]\}d\beta +\frac{N}{2}\,\log (2\pi {\sigma }^{2})$$

16 $$={\int }_{0}^{1}\,{\int }_{-\infty }^{\infty }\,NE({{\boldsymbol{\theta }}}_{k},\sigma ){\rm{P}}({{\boldsymbol{\theta }}}_{k}|\varepsilon ,\beta ,\sigma ,{M}_{k})d{{\boldsymbol{\theta }}}_{k}d\beta +\frac{N}{2}\,\log (2\pi {\sigma }^{2})\,$$

17 $$={\int }_{0}^{1}\,{\langle NE({{\boldsymbol{\theta }}}_{k},\sigma )\rangle }_{{\rm{P}}({{\boldsymbol{\theta }}}_{k}|{\boldsymbol{\varepsilon }},\beta ,\sigma ,{M}_{k})}d\beta +\frac{N}{2}\,\log (2\pi {\sigma }^{2}),\,$$

where $\langle \cdot \rangle$ represents an expectation and

18 $$P({{\boldsymbol{\theta }}}_{k}|{\boldsymbol{\varepsilon }},\beta ,\sigma ,{M}_{k})=\frac{\exp [\,-\,\beta NE({{\boldsymbol{\theta }}}_{k},\sigma )]{\rm{P}}({{\boldsymbol{\theta }}}_{k}|{M}_{k})}{{\int }_{-\infty }^{\infty }\,\exp [\,-\,\beta NE({{\boldsymbol{\theta }}}_{k},\sigma )]{\rm{P}}({{\boldsymbol{\theta }}}_{k}|{M}_{k})d{{\boldsymbol{\theta }}}_{k}}.$$

When $NE({{\boldsymbol{\theta }}}_{k},\sigma )$ is regarded as energy, Eq. (18) suggests that ${\rm{P}}({{\boldsymbol{\theta }}}_{k}|{\boldsymbol{\varepsilon }},\beta ,\sigma ,{M}_{k})$ and *β* correspond to the Boltzmann distribution and the inverse temperature in statistical physics, respectively. Equation (17) is approximated by a Riemann sum,

19 $$f({M}_{k},\sigma )\simeq \mathop{\sum }\limits_{l=1}^{L}\,{\langle NE({{\boldsymbol{\theta }}}_{k},\sigma )\rangle }_{{\rm{P}}({{\boldsymbol{\theta }}}_{k}|{\boldsymbol{\varepsilon }},{\beta }_{l},\sigma ,{M}_{k})}\Delta {\beta }_{l}+\frac{N}{2}\,\log (2\pi {\sigma }^{2}),$$

where ${\beta }_{l}$ is a sequence of inverse temperatures $0={\beta }_{1} < {\beta }_{2} < \cdots  < {\beta }_{L}=1$ obtained by dividing the region from $\beta =0$ to $\beta =1$ into $L$ pieces in some manner, and each ${\langle NE({{\boldsymbol{\theta }}}_{k},\sigma )\rangle }_{{\rm{P}}({{\boldsymbol{\theta }}}_{k}|{\boldsymbol{\varepsilon }},{\beta }_{l},\sigma ,{M}_{k})}$ is obtained by performing MCMC sampling independently at each inverse temperature ${\beta }_{l}$. However, MCMC sampling often results in trapping at local minima.

The replica exchange Monte Carlo (REMC) method is an algorithm of an MCMC method used to avoid trapping at local minima. The REMC method takes samples from the joint density

20 $${\rm{P}}({{\boldsymbol{\theta }}}_{k}^{1},{{\boldsymbol{\theta }}}_{k}^{2}\cdots {{\boldsymbol{\theta }}}_{k}^{L}|{\boldsymbol{\varepsilon }},\sigma ,{M}_{k})=\mathop{\prod }\limits_{l=1}^{L}\,{\rm{P}}({{\boldsymbol{\theta }}}_{k}^{l}|{\boldsymbol{\varepsilon }},{\beta }_{l},\sigma ,{M}_{k}),$$

where the probability density ${\rm{P}}({{\boldsymbol{\theta }}}_{k}^{l}|{\boldsymbol{\varepsilon }},{\beta }_{l},\sigma ,{M}_{k})$ is defined in Eq. (18). The REMC method performs sampling from the joint density ${\rm{P}}({{\boldsymbol{\theta }}}_{k}^{1},{{\boldsymbol{\theta }}}_{k}^{2}\cdots {{\boldsymbol{\theta }}}_{k}^{L}|{\boldsymbol{\varepsilon }},\sigma ,{M}_{k})$ on the basis of the following updates.

1. **Sampling from each density** ${\rm{P}}({{\boldsymbol{\theta }}}_{k}^{l}|{\boldsymbol{\varepsilon }},{\beta }_{l},\sigma ,{M}_{k})$
  Sampling ${{\boldsymbol{\theta }}}_{k}^{l}$ from ${\rm{P}}({{\boldsymbol{\theta }}}_{k}^{l}|{\boldsymbol{\varepsilon }},{\beta }_{l},\sigma ,{M}_{k})$ by a conventional MCMC method, such as the Metropolis– Hastings algorithm^20^.
2. **Exchange process between two densities corresponding to adjacent inverse temperatures**

The exchanges between the configurations ${{\boldsymbol{\theta }}}_{k}^{l}$ and ${{\boldsymbol{\theta }}}_{k}^{l+1}$ correspond to adjacent inverse temperatures following the probability $R=\,{\rm{\min }}(1,r)$, where

$$\begin{array}{rcl}r & = & \frac{{\rm{P}}({{\boldsymbol{\theta }}}_{k}^{1},\cdots ,{{\boldsymbol{\theta }}}_{k}^{l+1},{{\boldsymbol{\theta }}}_{k}^{l},\cdots ,{{\boldsymbol{\theta }}}_{k}^{L}|{\boldsymbol{\varepsilon }},\sigma ,{M}_{k})}{{\rm{P}}({{\boldsymbol{\theta }}}_{k}^{1},\cdots ,{{\boldsymbol{\theta }}}_{k}^{l},{{\boldsymbol{\theta }}}_{k}^{l+1},\cdots ,{{\boldsymbol{\theta }}}_{k}^{L}|{\boldsymbol{\varepsilon }},\sigma ,{M}_{k})}\\  & = & \frac{{\rm{P}}({{\boldsymbol{\theta }}}_{k}^{l+1}|{\boldsymbol{\varepsilon }},{\beta }_{l},\sigma ,{M}_{k}){\rm{P}}({{\boldsymbol{\theta }}}_{k}^{l}|{\boldsymbol{\varepsilon }},{\beta }_{l+1},\sigma ,{M}_{k})}{{\rm{P}}({{\boldsymbol{\theta }}}_{k}^{l}|{\boldsymbol{\varepsilon }},{\beta }_{l},\sigma ,{M}_{k}){\rm{P}}({{\boldsymbol{\theta }}}_{k}^{l+1}|{\boldsymbol{\varepsilon }},{\beta }_{l+1},\sigma ,{M}_{k})}\\  & = & \exp \{N[{\beta }_{l+1}-{\beta }_{l}][E({{\boldsymbol{\theta }}}_{k}^{l+1},\sigma )-E({{\boldsymbol{\theta }}}_{k}^{l},\sigma )]\}.\end{array}$$

Sampling from a distribution with a smaller $\beta$ corresponds to sampling from a distribution with a larger intensity of noise; thus, the distribution tends not to have a local minimum. Hence, sampling from the joint density ${\rm{P}}({{\boldsymbol{\theta }}}_{k}^{1},{{\boldsymbol{\theta }}}_{k}^{2}\cdots {{\boldsymbol{\theta }}}_{k}^{L}|{\boldsymbol{\varepsilon }},\sigma ,{M}_{k})$ overcomes the local minima in distriubtions with large $\beta$ and enables the rapid convergence of sampling.

Using the sampling result of the $\beta =1$ state, we can obtain the posterior distribution of the parameter ${\rm{P}}({{\boldsymbol{\theta }}}_{k}|{\boldsymbol{\varepsilon }},\sigma ,{M}_{k}):\,={\rm{P}}({{\boldsymbol{\theta }}}_{k}|{\boldsymbol{\varepsilon }},\beta =1,\sigma ,{M}_{k})$ [Eq. (18)] for the noise intensity $\sigma$. From the posterior distribution of ${{\boldsymbol{\theta }}}_{k}$, we can estimate the model parameters ${{\boldsymbol{\theta }}}_{k}$ of ${M}_{k}$ and the related information, such as the estimation accuracy.

From the sampling result of Eq. (20), $f({M}_{k},{\sigma }_{s})$ can be obtained, where ${\sigma }_{s}:=\sqrt{\frac{1}{{\beta }_{s}}}\sigma$. Here, we describe how to obtain $f({M}_{k},{\sigma }_{s})$. $f({M}_{k},{\sigma }_{s})$ can be rewritten by using $\sigma$ as

21 $$f({M}_{k},{\sigma }_{s})={\int }_{0}^{1}\,{\langle NE({{\boldsymbol{\theta }}}_{k},{\sigma }_{s})\rangle }_{{\rm{P}}({{\boldsymbol{\theta }}}_{k}|{\boldsymbol{\varepsilon }},\beta {\prime} ,{\sigma }_{s},{M}_{k})}d\beta {\prime} +\frac{N}{2}\,\log (2\pi {\sigma }_{s}^{2})\,$$

22 $$\,={\int }_{0}^{\frac{{\sigma }^{2}}{{\sigma }_{s}^{2}}}\,{\langle NE({{\boldsymbol{\theta }}}_{k},\sigma )\rangle }_{{\rm{P}}({{\boldsymbol{\theta }}}_{k}|{\boldsymbol{\varepsilon }},\beta ,\sigma ,{M}_{k})}\frac{{\sigma }_{s}^{2}}{{\sigma }^{2}}d\beta +\frac{N}{2}\,\log (2\pi {\sigma }_{s}^{2}),$$

where $\beta {\prime} =\frac{{\sigma }_{s}^{2}}{{\sigma }^{2}}\beta$. Then, $f({M}_{k},{\sigma }_{s})$ can be obtained from a Riemann sum as

23 $$f({M}_{k},{\sigma }_{s})\simeq \frac{{\sigma }_{s}^{2}}{{\sigma }^{2}}\,\mathop{\sum }\limits_{l=0}^{s}\,{\langle NE({{\boldsymbol{\theta }}}_{k},\sigma )\rangle }_{{\rm{P}}({{\boldsymbol{\theta }}}_{k}|{\boldsymbol{\varepsilon }},{\beta }_{l},\sigma ,{M}_{k})}\Delta {\beta }_{l}+\frac{N}{2}\,\log (2\pi {\sigma }_{s}^{2}).$$

Thus, it is possible to calculate an approximate value of $f({M}_{k},{\sigma }_{s})$ using the obtained expectation values ${\langle NE({{\boldsymbol{\theta }}}_{k},\sigma )\rangle }_{{\rm{P}}({{\boldsymbol{\theta }}}_{k}|{\boldsymbol{\varepsilon }},{\beta }_{l},\sigma ,{M}_{k})}$ by sampling at $\sigma$. Using the obtained integral value set ${\{f({M}_{k},{\sigma }_{s})\}}_{s=1}^{s=L}$, the Bayesian free energy $F({M}_{k})$ can be calculated as

24 $$F({M}_{k})=-\,\log \,{\int }_{-\infty }^{\infty }\,d\sigma {\rm{P}}({\boldsymbol{\varepsilon }}|\sigma ,{M}_{k})P(\sigma )\,$$

25 $$\,=-\,\log \,{\int }_{-\infty }^{\infty }\,d\sigma \,\exp [-f({M}_{k},\sigma )]P(\sigma )$$

26 $$\,\simeq -\,\log \,\mathop{\sum }\limits_{s=1}^{L}\,\exp [-f({M}_{k},{\sigma }_{s})]P({\sigma }_{s})\Delta {\sigma }_{s}\mathrm{}.$$

## Application example

Here, as an application example to verify the effectiveness of the proposed framework, we evaluate the Kimura model, which is one of the most successful creep constitutive equations, and the modified Kimura model, which was created as a model for comparison with the Kimura model. These models are difficult to evaluate using the existing framework.

### Creep constitution models and material

**Figure 2:**
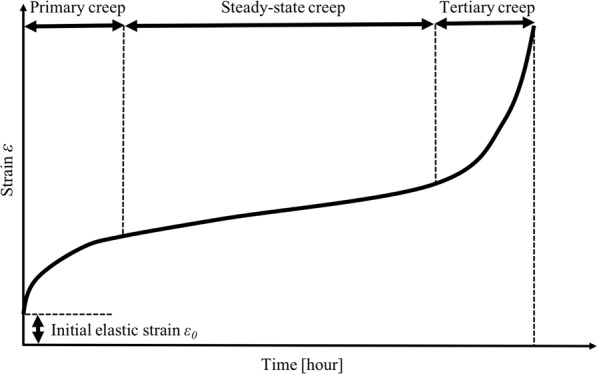
Creep curves and three time domains. Primary creep represents the zone of creep rate deceleration, steady-state creep represents the creep rate zone of constant velocity, and tertiary creep represents the final acceleration zone.

The modified Kimura model was set on the basis of the following assumptions. There are a number of creep constitutive models, which are roughly classified into two types according to the way the steady state, where the deformation rate is constant, is modeled (Fig. 2). One is a steady-state creep model, in which a linear region with a constant deformation rate is represented as an independent linear term. The other is the unsteady creep model, in which the steady state is generated by the balance between the deceleration of the primary creep and the acceleration of the tertiary creep. The Kimura model is formulated as Eq. (1). This model is an unsteady creep constitutive equation in which ${a}_{1}{t}^{{b}_{1}}+{a}_{2}{t}^{{b}_{2}}$ represents the primary creep and ${c}_{1}\,\exp ({d}_{1}t)+{c}_{2}\,\exp ({d}_{2}t)$ represents the tertiary creep, and the linear region is represented by its balance. By regression analysis using the creep data set of Gr.91 steel, it was previously found that ${b}_{2}$ takes a value close to 1^1^. This implies that the Kimura model models the steady state as a linear term rather than a balance. In our previous study^13^, it was also found that, using the measurement data^14^ of Gr.91 steel, the likelihood of another type of creep constitutive model, the theta projection model^4^, was improved by adding a linear term. On the other hand, it was also reported by Kimura *et al*. that ${b}_{2}$ can take a value larger than 1 by changing the fitting method^2^. To determine whether a steady state is required, we designed the following steady-state creep model by replacing ${b}_{2}$ in the Kimura model with 1.

27 $${\boldsymbol{\varepsilon }}={{\boldsymbol{\varepsilon }}}_{0}+{a}_{1}{t}^{{b}_{1}}+{a}_{2}t+{c}_{1}\,\exp ({d}_{1}t)+{c}_{2}\,\exp ({d}_{2}t)$$

We call this regression model the modified Kimura model. Using the proposed framework, we determined whether the modified Kimura model or the Kimura model is more likely for Gr.91 creep data (Table 1) obtained at 650 °C and 80 MPa.

**Table 1:** Chemical composition of the Gr.91 steel (9Cr-1Mo-Nb-V)^14^.

	C	Si	Mn	P	S	Cu	Ni	Cr	Mo	V	Nb	Al	N
(mass%)	0.10	0.25	0.43	0.006	0.002	0.012	0.06	8.87	0.93	0.19	0.07	0.014	0.06

In this verification, on the basis of the regression results shown in refs. ^1^ and^2^, the prior distributions of the Kimura model and the modified Kimura model were set as shown in Table 2. In Bayesian inference, priors are an important part of the model. To examine the effect of the prior distribution in Bayesian model selection, several different prior distributions of the noise intensity $\sigma$ were prepared. The relationship between the prior and the model selection result was verified. Concretely, the prior distribution of the noise intensity $\sigma$ was set as a uniform distribution with a certain value as the lower limit and 10 times the lower limit as the upper limit. Then, 100 different prior distributions were prepared with values of the lower limit from $3.0\times {10}^{-5}$ to $3.3\times {10}^{-3}$ at equal intervals in logarithmic space. We applied the proposed framework under these conditions.

**Table 2:** Prior distributions of Kimura model and modified Kimura model.

param	min	max	param	min	max
*a*_1_	1.0 × 10^−4^	2.0 × 10^−3^	*b*_1_	1.0 × 10^−1.0^	1.0 × 10^0.0^
*a*_2_	1.0 × 10^−7^	2.0 × 10^−5^	*b*_2_	0.5	1.9
*c*_1_	1.0 × 10^−5^	3.0 × 10^−4^	*d*_1_	1.0 × 10^−3.0^	1.0 × 10^−2.5^
*c*_2_	1.0 × 10^−22^	5.0 × 10^−15^	*d*_2_	1.0 × 10^−3.0^	1.0 × 10^−1.9^
*ε*_0_	1.0 × 10^−4^	1.0 × 10^−3^	*σ*	3.0 × 10^−5^ to 3.3 × 10^−3^	3.0 × 10^−4^ to 3.3 × 10^−2^

All prior distributions were uniform, and their upper and lower limits were set as follows. The prior distribution of *b*_2_ was used only with the Kimura model.

When we set the prior distributions of ${a}_{1}$, ${a}_{2}$, ${b}_{1}$, ${b}_{2}$, ${c}_{1}$, ${c}_{2}$, ${d}_{1}$, and ${d}_{2}$ as described in Table 2, the exchange symmetry of parameter pairs in the Kimura model disappears. The exchange symmetry is one of the reasons why the Kimura model is an undetermined model. On the other hand, the Kimura model continues to have an undetermined structure around the parameters estimated as the maximum-likelihood solution for the following reasons. To model the transition of a creep curve under a change in stress and temperature conditions, Kimura *et al*. estimated the stress and temperature dependences of parameters. They reported that the distribution of ${c}_{2}$ in stress and temperature space has a significantly wider dispersion than the other parameters^1,2^. This behavior can be understood from the fact that the estimation of parameter ${c}_{2}$ is unstable, and, therefore, the parameters of the Kimura model are not well-determined around the maximum-likelihood solution.

In the REMC sampling, we adopted the Metropolis– Hastings algorithm^20^ to sample each state of the inverse temperature. The states of the inverse temperature were determined using the following exponential function^21^:

28 $${\beta }_{l}=\{\begin{array}{ll}0.0 & (l=1)\\ {\gamma }^{l-L} & (l\ge 13)\end{array},$$

where $L=311$ and $\gamma =1.05$. The approximate error $E{r}_{l}$ of the numerical integration, i.e., the Riemann sum, between ${\beta }_{l}$ and ${\beta }_{l+1}$ is given as

29 $$E{r}_{l}\sim \frac{1}{2}[{\langle NE({{\boldsymbol{\theta }}}_{k},\sigma )\rangle }_{{\rm{P}}({{\boldsymbol{\theta }}}_{k}|{\boldsymbol{\varepsilon }},{\beta }_{l},\sigma ,{M}_{k})}-{\langle NE({{\boldsymbol{\theta }}}_{k},\sigma )\rangle }_{{\rm{P}}({{\boldsymbol{\theta }}}_{k}|{\boldsymbol{\varepsilon }},{\beta }_{l+1},\sigma ,{M}_{k})}]\Delta {\beta }_{l}$$

30 $$\,=\frac{1}{2}\Delta {\langle E\rangle }_{l}\Delta {\beta }_{l}.$$

Therefore, it is necessary to make the division $\Delta {\beta }_{l}$ sufficiently smaller than $\Delta {\langle E\rangle }_{l}$. In the Kimura model, which is a linear sum of exponential functions and power functions, slight parameter variations cause large functional changes. In particular, at $l=1$, where sampling is performed over the entire range of the prior distribution, the $l=1$ state takes a very large average energy $\langle {E}_{1}\rangle$ because of such the nature of this model. On the other hand, in the region of $l > 1$, the parameter region with a small squared error is sampled. Therefore, the energy in the $l=2$ state becomes $\langle {E}_{2}\rangle \ll \langle {E}_{1}\rangle$. As a result, $\Delta {\langle E\rangle }_{1}$ becomes very large and the approximation error $E{r}_{1}$ becomes non-negligible. Therefore, in this study, to reduce the approximation error $E{r}_{1}$, the following temperature states are inserted between ${\beta }_{1}$ and ${\beta }_{13}$ so that $\Delta {\beta }_{1} < \Delta {\langle E\rangle }_{1}$.

31 $${\beta }_{l}=\{\begin{array}{ll}{10}^{-28} & (l=2)\\ {10}^{-25} & (l=3)\\ {10}^{-22} & (l=4)\\ {10}^{-20} & (l=5)\\ {10}^{-18} & (l=6)\\ {10}^{-16} & (l=7)\\ {10}^{-14} & (l=8)\\ {10}^{-12} & (l=9)\\ {10}^{-10} & (l=10)\\ {10}^{-8} & (l=11)\\ {10}^{-7} & (l=12)\end{array}$$

In the sampling, we abandoned the first 100,000 steps and sampled the next 100,000 steps.

## Results

In this study, model parameters were estimated from the posterior distributions using the maximum a posteriori (MAP) method. The MAP method estimates parameters on the basis of the following equations:

32 $$\begin{array}{rcl}{\sigma }^{MAP} & = & \mathop{{\rm{\arg }}\,{\rm{\max }}}\limits_{{\sigma }_{l}}\,{\rm{P}}({\sigma }_{l}|{\boldsymbol{\varepsilon }},{M}_{k}),\\ {{\boldsymbol{\theta }}}_{k}^{MAP} & = & \mathop{{\rm{\arg }}\,{\rm{\max }}}\limits_{{{\boldsymbol{\theta }}}_{k}}\,{\rm{P}}({{\boldsymbol{\theta }}}_{k}|{\boldsymbol{\varepsilon }},{\sigma }^{MAP},{M}_{k}).\end{array}$$

**Figure 3:**
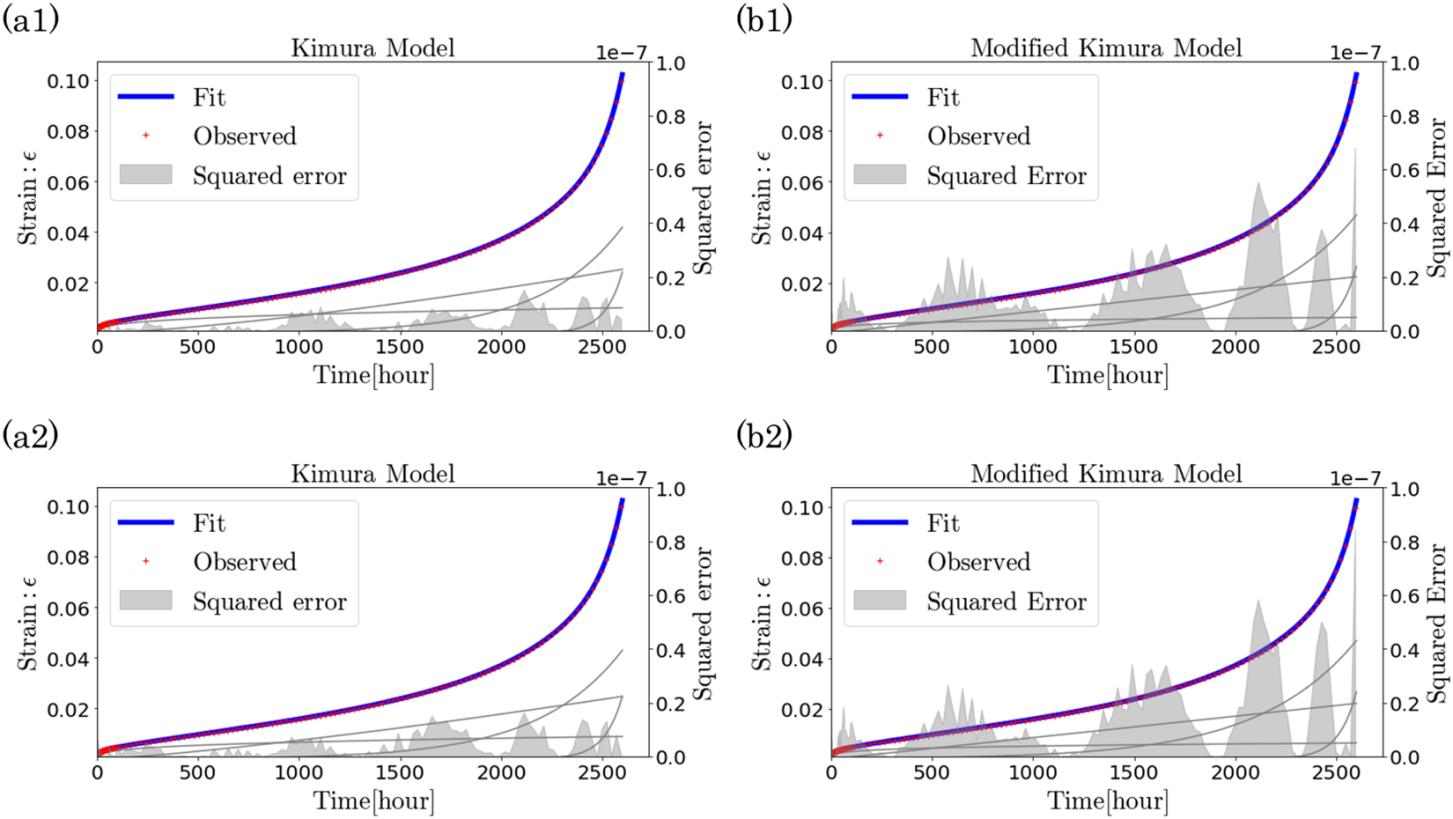
Fitting results (blue curve) of creep strain data (red points). (**a1**,**b1**) Fitting results of Kimura model (**a1**) and modified Kimura model (**b1**) with noise prior set as a uniform distribution from $4.0\times {10}^{-5}$ to $4.0\times {10}^{-4}$. (**a2**,**b2**) Fitting results of Kimura model (**a2**) and modified Kimura model (**b2**) with noise prior set as a uniform distribution from $4.0\times {10}^{-4}$ to $4.0\times {10}^{-3}$. The gray curve represents the component of each term included in the Kimura and modified Kimura models, and the gray area represents the squared error between the experimental data and the regression curve at each time.

We examined the fitting results by the MAP solution with two types of noise prior. One was a small-noise-intensity prior, which was set as a uniform distribution from $4.0\times {10}^{-5}$ to $4.0\times {10}^{-4}$, and the other was a large-noise-intensity prior, which was set as a uniform distribution from $4.0\times {10}^{-4}$ to $4.0\times {10}^{-3}$. From the regression results (Fig. 3), it was confirmed that both the Kimura model and the modified Kimura model provided a good fitting regardless of the prior distribution of noise. To examine the regression results more precisely, we compared the mean square error (MSE) for the fitting results:

33 $$MSE=\frac{1}{N}\,\mathop{\sum }\limits_{i=1}^{N}\,{({{\boldsymbol{\varepsilon }}}_{i}-{{\boldsymbol{\varepsilon }}}_{k}({t}_{i};{{\boldsymbol{\theta }}}_{k}))}^{2}.$$

**Figure 4:**
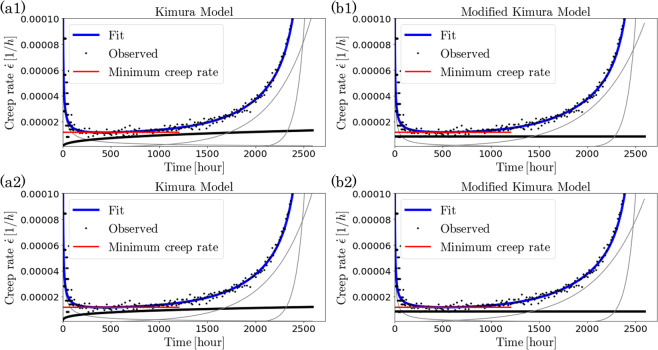
Creep rate curves obtained by differentiating the regression strain curve. (**a1**,**b1**) Creep rate curve obtained by fitting strain curve by Kimura model (**a1**) and modified Kimura model (**b1**) with noise prior set as uniform distribution from $4.0\times {10}^{-5}$ to $4.0\times {10}^{-4}$. (**a2**,**b2**) Creep rate curve obtained by fitting strain curve by Kimura model (**a2**) and modified Kimura model (**b2**) with noise prior set as uniform distribution from $4.0\times {10}^{-4}$ to $4.0\times {10}^{-3}$. The creep rate data points (black dots) were calculated as the slope of the adjacent points of strain measurement data. The red line represents the minimum creep rate estimated by the creep measurement experimenter^13^. The gray and black lines represent the components of each term in the model. In particular, the black line represents the component corresponding to the second term of the model.

For the small-noise-intensity prior distribution, the MSE of the Kimura model was $1.65\times {10}^{-9}$ and that of the modified Kimura model was $8.31\times {10}^{-9}$. For the large-noise-intensity prior distribution, the MSE of the Kimura model was $2.69\times {10}^{-9}$ and that of the modified Kimura model was $8.44\times {10}^{-9}$. Thus, the Kimura model always has a smaller MSE regardless of the noise intensity of the prior distribution. Moreover, the MSE did not change significantly with the noise intensity of the prior distribution. It was also confirmed that the squared error between the experimental data and the regression curve at each time (gray area of Fig. 3) has the same error distribution in each model regardless of the noise intensity. The creep rate curve was calculated by differentiating this regression curve. The estimated creep rate curves closely fit the creep rate data points. The creep rate data points was estimated as the difference in the adjacent points of strain data (black dots in Fig. 4). The value of the coefficient ${a}_{2}$ of the modified Kimura model [Eq. (27)], representing stationary creep, was similar to the minimum creep rate estimated by the experimenter as the creep rate in the steady area^13^. This result is consistent with the assumption that the second term of the modified Kimura model models the steady-state creep.

**Figure 5:**
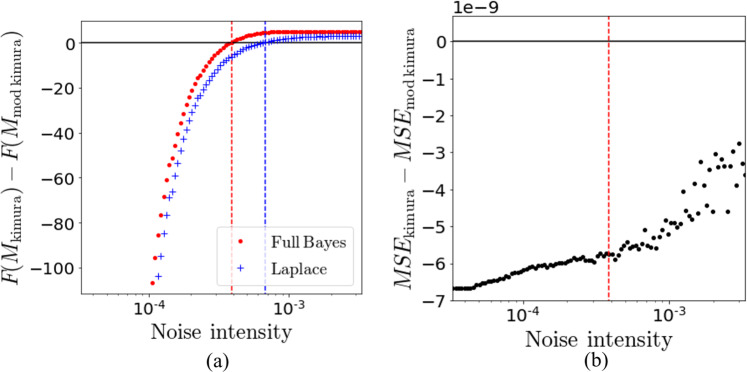
(**a**) Model selection result. Relationship between prior distribution of noise intensity *σ* and model selection results of Kimura model and modified Kimura model. The x-axis represents the lower bound of the prior distribution of noise intensity. The prior distribution was a uniform distribution from this value to 10 times this value. “Full Bayes” is the result of the proposed framework and “Laplace” is the result of the existing framework proposed by Keitel *et al*.^17^. (**b**) Comparison of MSE of Kimura model and modified Kimura model. The x-axis is the same as in (**a**).

Using the proposed framework, it was confirmed that the selected model switched from the Kimura model to the modified Kimura model at the point where the prior distribution of the noise intensity $\sigma$ is set as a uniform distribution from $3.88\times {10}^{-4}$ to $3.88\times {10}^{-3}$ (Fig. 5(a)). On the other hand, it was also confirmed that the MSE of the Kimura model was always smaller than that of the modified Kimura model regardless of the noise prior distribution (Fig. 5(b)). For comparison with the existing framework^17^, model evaluation based on the existing framework was performed. In the existing framework^17^, the Bayesian free energy is calculated on the basis of the Laplace approximation (see Appendix A for a more general theory of the Laplace approximation including *σ*). Using the MAP solution ${{\boldsymbol{\theta }}}_{k}^{MAP}$, the Laplace approximation is given by

34 $$\begin{array}{rcl}F({M}_{k}) & \sim  & -\log \,{\rm{P}}({\boldsymbol{\varepsilon }}|{{\boldsymbol{\theta }}}_{k}^{MAP},{\sigma }^{MAP},{M}_{k}){\rm{P}}({{\boldsymbol{\theta }}}_{k}^{MAP}|{M}_{k}){\rm{P}}({\sigma }^{MAP}|{M}_{k})\\  &  & -\,\frac{d+1}{2}\,\log (2\pi )+\frac{d+1}{2}\,\log (N)+\frac{1}{2}\,\log [{\rm{\det }}({\bf{H}})],\end{array}$$

where *H* is the Hessian matrix: ${{\bf{H}}}^{ij}={\sum }_{i=1}^{d+1}\,{\sum }_{j=1}^{d+1}\,{\frac{{\partial }^{2}g({{\boldsymbol{\theta }}}_{k})}{\partial {{\boldsymbol{\theta }}}_{k}^{i}\partial {{\boldsymbol{\theta }}}_{k}^{j}}|}_{{{\boldsymbol{\theta }}}_{k}={{\boldsymbol{\theta }}}_{k}^{MAP}}$. In the existing framework^17^, *H* is replaced by the inverse of the variance-covariance matrix ${{\bf{Cov}}}^{-1}$ of *θ*_*k*_ to reduce the computational cost,

35 $$\begin{array}{rcl}F({M}_{k}) & \sim  & -\log \,{\rm{P}}({\boldsymbol{\varepsilon }}|{{\boldsymbol{\theta }}}_{k}^{MAP},{\sigma }^{MAP},{M}_{k}){\rm{P}}({{\boldsymbol{\theta }}}_{k}^{MAP}|{M}_{k}){\rm{P}}({\sigma }^{MAP}|{M}_{k})\\  &  & -\,\frac{d+1}{2}\,\log \,\mathrm{(2}\pi )+\frac{d+1}{2}\,\log (N)-\frac{1}{2}\,\log [{\rm{\det }}({\bf{Cov}})]\mathrm{}.\end{array}$$

Model selection was performed using this approximated free energy. In the model selection results using this approximated free energy, selected models are switched at a higher noise intensity than the switched noise intensity of the proposed framework (Fig. 5(a)). This means that the existing framework more often evaluates the Kimura model as the more likely model than the proposed framework.

To visualize a posterior distribution with three or more dimensions, we calculated the following marginal posterior distribution, which marginalizes the posterior distribution of the parameters ${{\boldsymbol{\theta }}}_{k}^{\neg m}$ except for the parameter of interest ${{\boldsymbol{\theta }}}_{k}^{m}$:

36 $${\rm{P}}({{\boldsymbol{\theta }}}_{k}^{m}|{\boldsymbol{\varepsilon }},{\sigma }^{MAP},{M}_{k})={\int }_{-\infty }^{\infty }\,{\rm{P}}({{\boldsymbol{\theta }}}_{k}|{\boldsymbol{\varepsilon }},{\sigma }^{MAP},{M}_{k}){\rm{P}}({{\boldsymbol{\theta }}}_{k},{M}_{k})d{{\boldsymbol{\theta }}}_{k}^{\neg m}.$$

**Figure 6:**
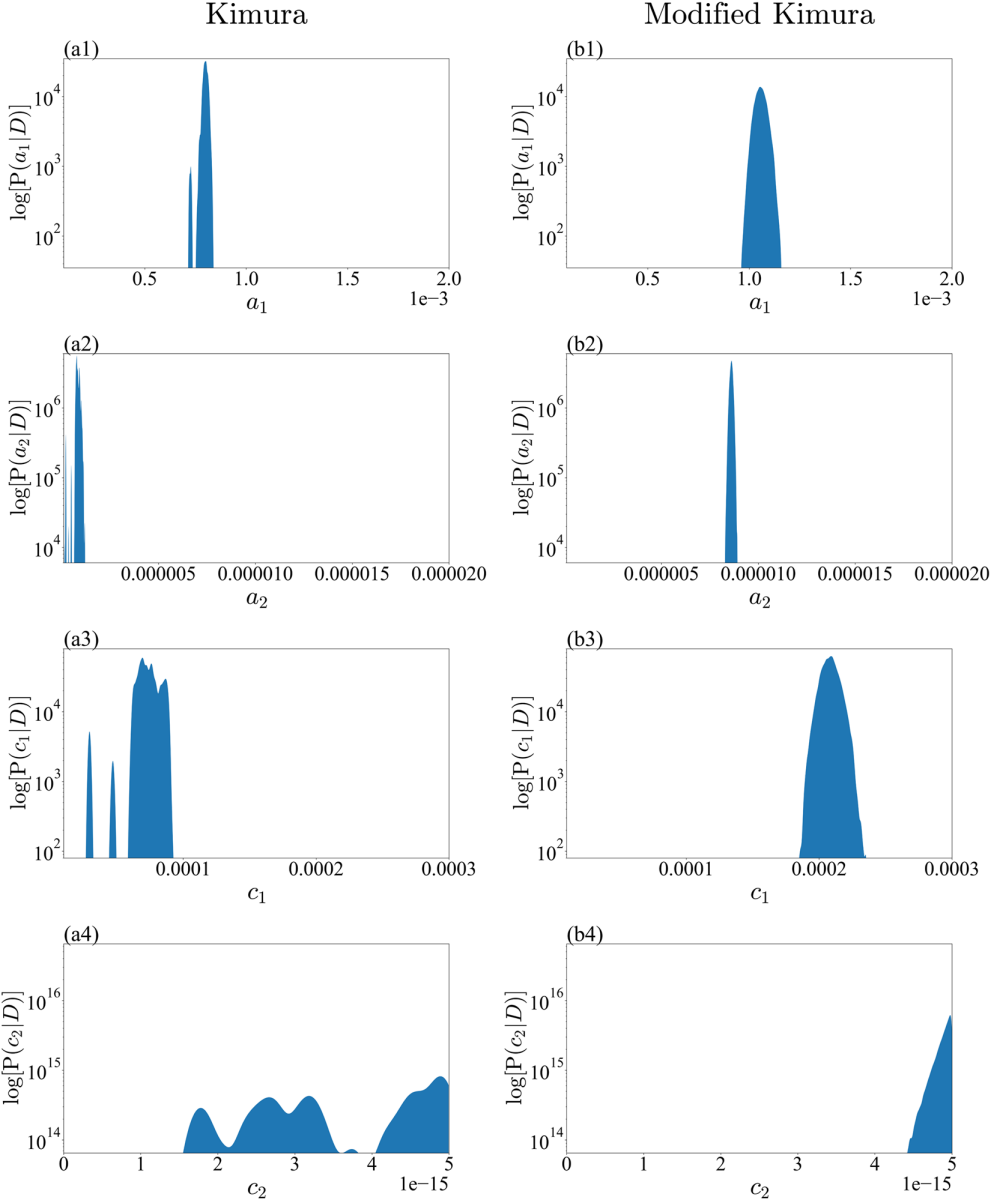
Marginalized posterior distributions for model parameters $\{{a}_{1},{a}_{2},{c}_{1},{c}_{2}\}$ of Kimura model and modified Kimura model with small-noise-intensity prior. (**a1**) ${a}_{1}$ of Kimura model. (**b1**) ${a}_{1}$ of modified Kimura model. (**a2**) ${a}_{2}$ of Kimura model. (**b2**) ${a}_{2}$ of modified Kimura model. (**a3**) ${c}_{1}$ of Kimura model. (**b3**) ${c}_{1}$ of modified Kimura model. (**a4**) ${c}_{2}$ of Kimura model. (**a4**) ${c}_{2}$ of modified Kimura model.

**Figure 7:**
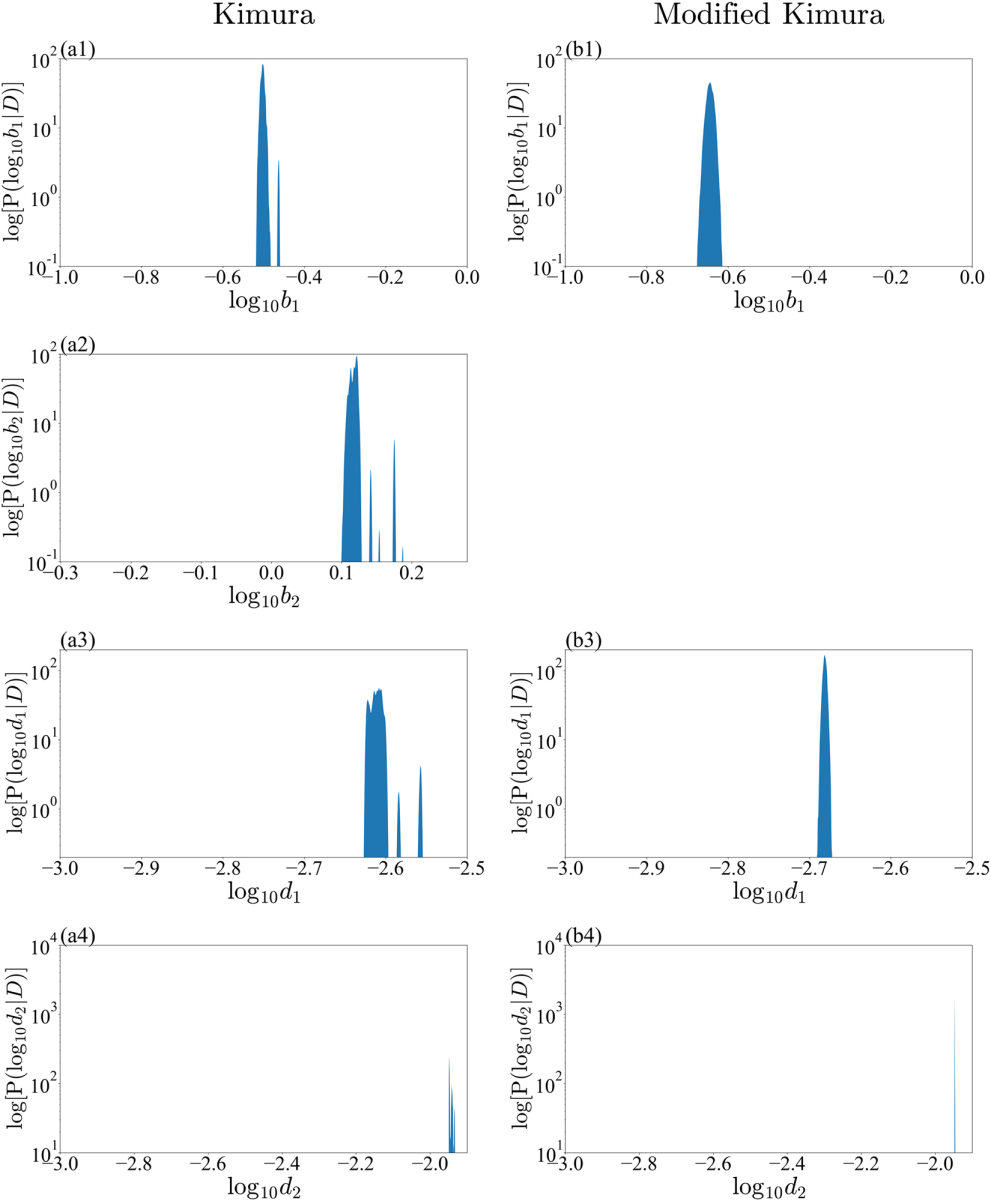
Marginalized posterior distributions for model parameters $\{{b}_{1},{b}_{2},{d}_{1},{d}_{2}\}$ of Kimura model and modified Kimura model with small-noise-intensity prior. (**a1**) ${b}_{1}$ of Kimura model. (**b1**) ${b}_{1}$ of modified Kimura model. (**a2**) ${b}_{2}$ of Kimura model. (**a3**) ${d}_{1}$ of Kimura model. (**b3**) ${d}_{1}$ of modified Kimura model. (**a4**) ${d}_{2}$ of Kimura model. (**a4**) ${d}_{2}$ of modified Kimura model.

**Figure 8:**
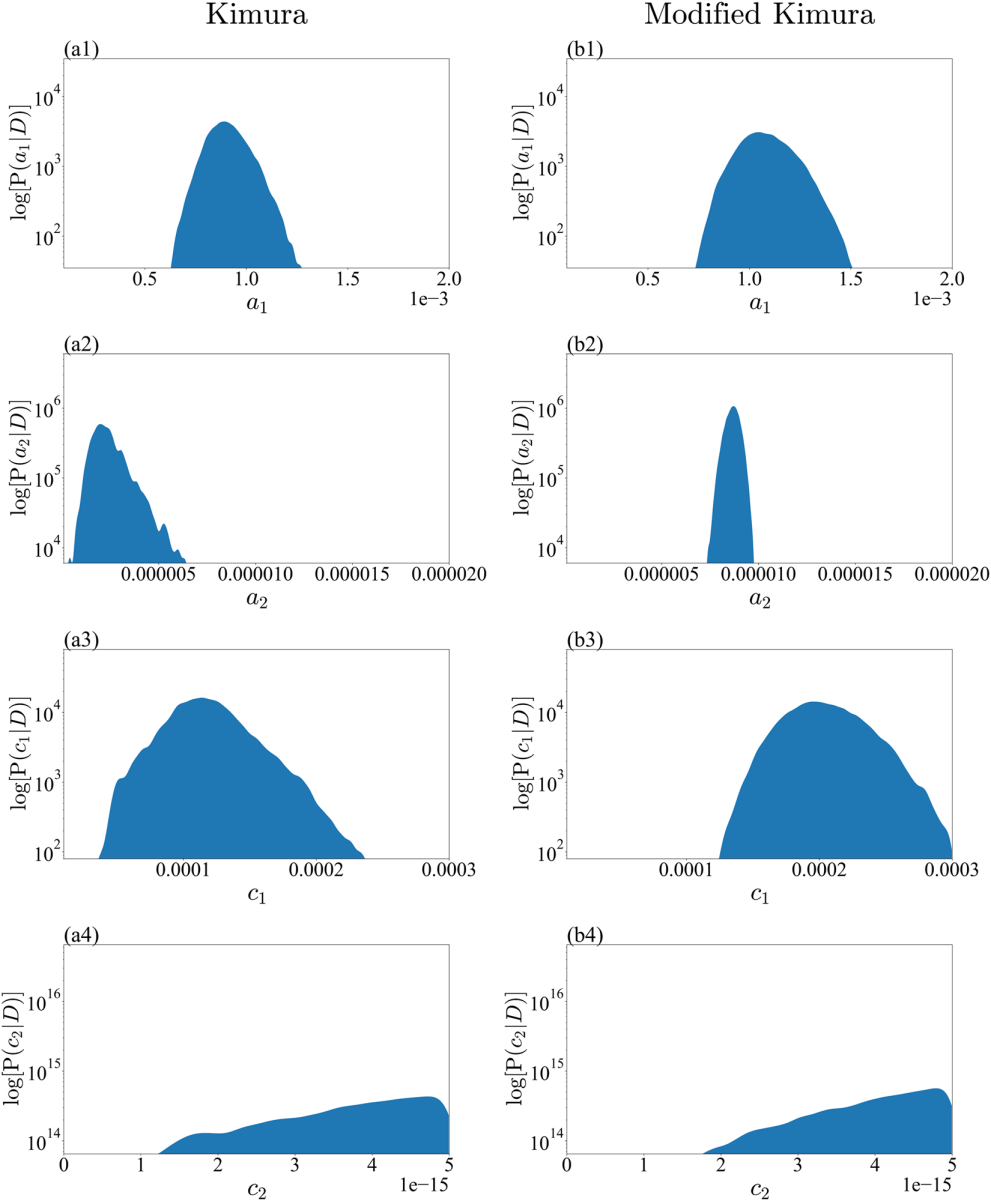
Marginalized posterior distributions for model parameters $\{{a}_{1},{a}_{2},{c}_{1},{c}_{2}\}$ of Kimura model and modified Kimura model with large-noise-intensity prior. (**a1**) ${a}_{1}$ of Kimura model. (**b1**) ${a}_{1}$ of modified Kimura model. (**a2**) ${a}_{2}$ of Kimura model. (**b2**) ${a}_{2}$ of modified Kimura model. (**a3**) ${c}_{1}$ of Kimura model. (**b3**) ${c}_{1}$ of modified Kimura model. (**a4**) ${c}_{2}$ of Kimura model. (**a4**) ${c}_{2}$ of modified Kimura model.

**Figure 9:**
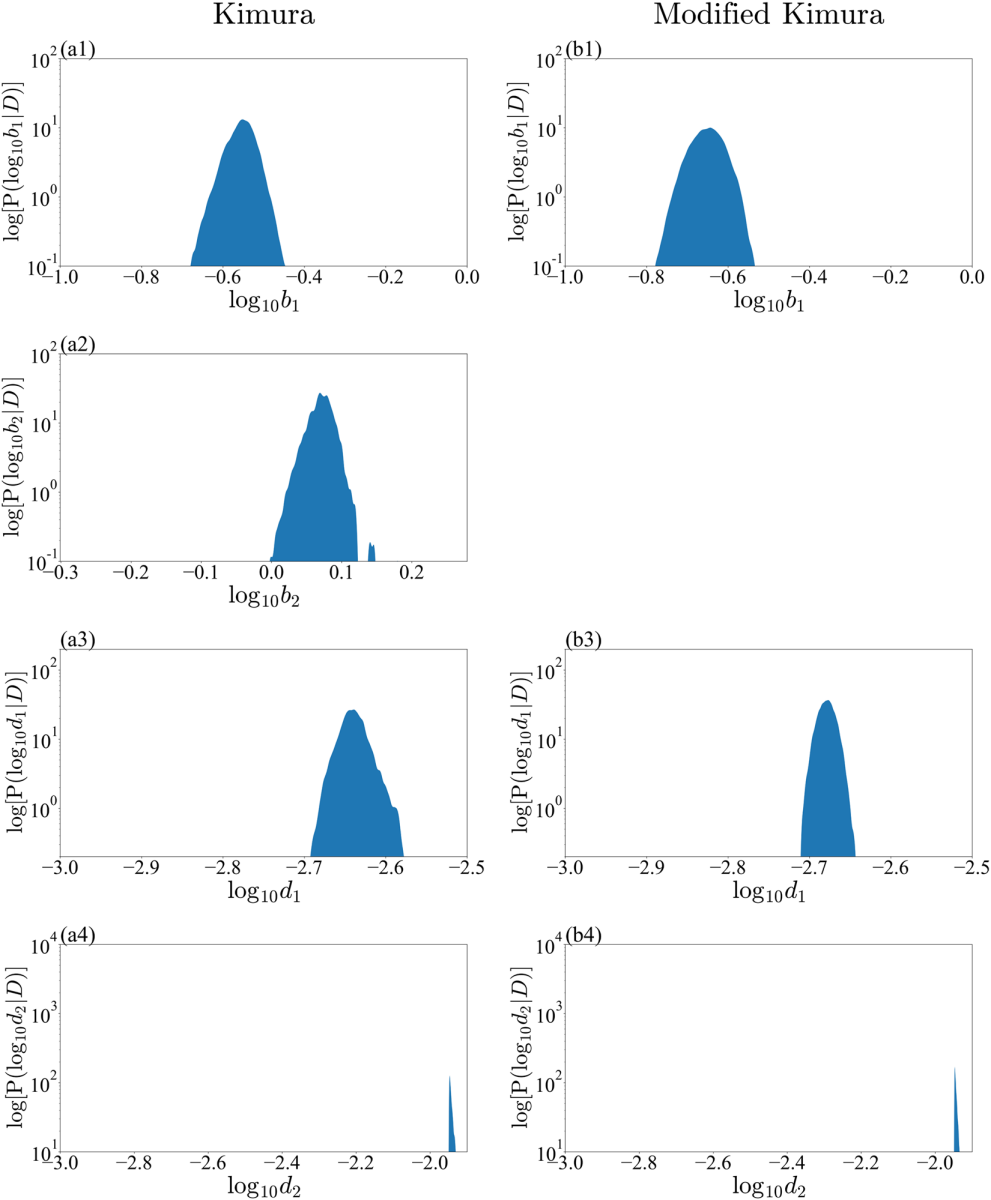
Marginalized posterior distributions for model parameters $\{{b}_{1},{b}_{2},{d}_{1},{d}_{2}\}$ of Kimura model and modified Kimura model with large-noise-intensity prior. (**a1**) ${b}_{1}$ of Kimura model. (**b1**) ${b}_{1}$ of modified Kimura model. (**a2**) ${b}_{2}$ of Kimura model. (**a3**) ${d}_{1}$ of Kimura model. (**b3**) ${d}_{1}$ of modified Kimura model. (**a4**) ${d}_{2}$ of Kimura model. (**a4**) ${d}_{2}$ of modified Kimura model.

**Figure 10:**
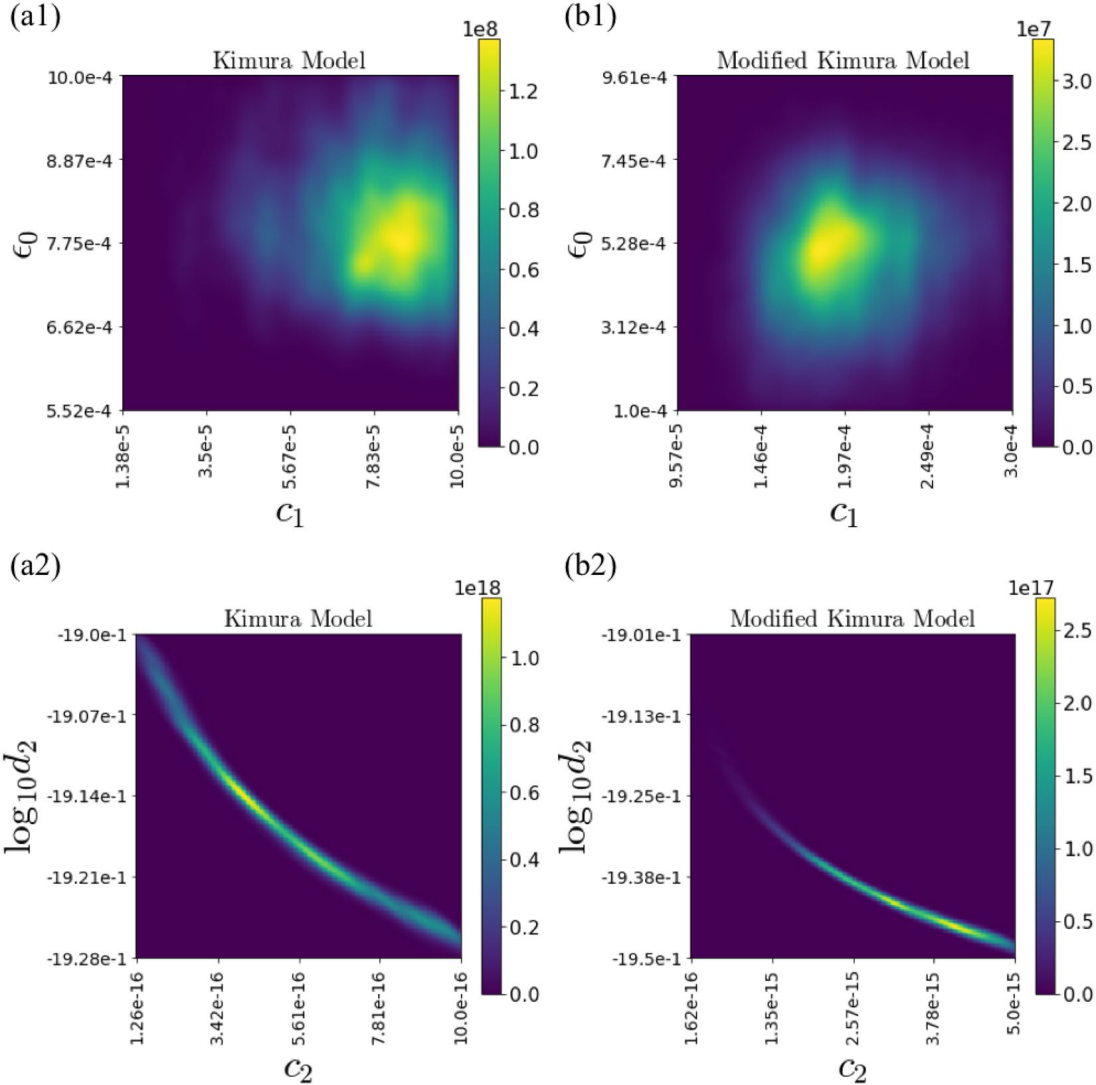
Marginalized posterior distributions for two parameters.

This integration can be approximated from the sum of the sampling result of the REMC method. The density function of the marginal posterior can be estimated by the kernel density estimation method using Gaussian kernels. We determined the bandwidth of the Gaussian kernels using Scott’s rule^22^. We compared the marginalized posterior distribution focusing on one parameter for the small-noise-intensity prior, where the Kimura model was selected (Figs. 6 and 7), and the large-noise-intensity prior, where the modified Kimura model was selected (Figs. 8 and 9). From the visualization results of the marginalized posterior distribution for the small-noise-intensity prior (Figs. 6 and 7), it was confirmed that the parameters’ posterior distribution of the Kimura model has a multimodal and complex structure, which is different from the posterior distribution of the modified Kimura model. The posterior distribution of ${c}_{2}$ with a small-noise-intensity prior has a particularly multimodal and broadened distribution (Fig. 6(a4)). Whereas, in the large-noise-intensity prior, it was observed that the posterior distribution of the Kimura model also became unimodal distribution (Figs. 8 and 9). Next, we examined the relationship between two parameters from the marginalized posterior distribution. Many marginalized posterior distributions were unstructured isotropic Gaussian distributions such as the marginalized posterior distributions of ${c}_{1}$ and ${{\boldsymbol{\varepsilon }}}_{0}$ (Figs. 10(a1,b1)). On the other hand, the marginalized posterior distributions between parameters of the exponential part and the coefficient part of the basis function have structured distributions, such as ${c}_{2}$ and ${\log }_{10}\,{d}_{2}$ (Figs. 10(a2,b2)).

## Summary and Discussion

The results of the application of the proposed framework to the Kimura model show that the model selection results vary depending on the noise intensity range. Furthermore, it is clarified for the first time that the posterior distribution of the Kimura model is multimodal in the small-noise-intensity region. In this section, after discussing the validity of the model selection results and their properties, we compare the Kimura model and the modified Kimura model in terms of multimodality of them posterior distributions.

Kimura *et al*. achieved high-accuracy fitting to creep measurement data using the Kimura model^1,2^. Similarly to their work^1,2^, our proposed framework achieved data fitting with high accuracy. This suggests that our framework performed under the same conditions as the Kimura model. In addition, the closeness of the minimum creep rate and the slope ${a}_{2}$ of the second term of the modified Kimura model (Eq. (27)) suggests that the steady state was modeled by the linear term ${a}_{2}t$, which was assumed when we set the modified Kimura model. Thus, it was confirmed that the regression results using the MAP solution of the proposed framework were reasonable.

The MSE of the Kimura model was smaller than that of the modified Kimura model, even in the large-noise-intensity prior distribution, in which the modified Kimura model was selected. This result indicates that the proposed framework gives model selection results that do not depend solely on the magnitude of the error. The results are explained using the following approximated Bayesian free energy formula. By extracting terms higher than order $log(N)$ from the result of the Laplace approximation, the following equation is obtained (see Appendix A):

37 $$F({M}_{k})\sim -\,\log \,{\rm{P}}({\boldsymbol{\varepsilon }}|{{\boldsymbol{\theta }}}_{k}^{MAP},{\sigma }^{MAP},{M}_{k})+\frac{d+1}{2}\,\log (N)$$

38 $$\,=NE({{\boldsymbol{\theta }}}_{k}^{MAP},\sigma )+\frac{d+1}{2}\,\log (N),$$

where *d* is the number of model parameters ${{\boldsymbol{\theta }}}_{k}$. The first term in Eq. (38) represents the regression error of the model and the second term represents the complexity of the model. In general, the more complex the model, the smaller the value of the first term; however, overfitting occurs in more complex models. The presence of the second term in the Bayesian free energy makes it possible to evaluate the model while preventing overfitting.

Thus, the Laplace approximation is useful for a rough clarification of the property of Bayesian free energy. However, the Laplace approximation is based on the hypothesis that the posterior distribution of the parameters is a unimodal Gaussian distribution. On the other hand, the proposed method selects the model by assuming that the posterior distributions of the Kimura model with small-noise-intensity prior are multimodal. The difference in the model selection results between the frameworks (Fig. 5(a)) is due to this difference in the property of the Laplace approximation and the proposed framework. This presumption is validated from the behavior of $\Delta F=F({M}_{{\rm{kimura}}})-F({M}_{{\rm{mod}}{\rm{kimura}}})$ in the Laplace approximation and the proposed framework (Fig. 5(a)), which tends to be small in a large-noise-intensity prior situation where the posterior distribution of the Kimura model is estimated as unimodal in the proposed framework (Figs. 8(a1–4) and 9(a1–4)). This might be the mechanism by which the switching noise intensity of the selected model differs between the proposed framework and the Laplace approximation (Fig. 5(a)).

Using the proposed framework, it is possible to set the prior knowledge of the measurement noise range as the prior distribution. In this research, we developed an efficient marginalization method of the noise intensity $\sigma$ using the sampling result of the REMC method. Such a method is novel not only in creep model selection but also in general Bayesian model selection. As a result of the model selection with this prior noise distribution, it was confirmed that the model selection results of the creep constitutive equation markedly change with the prior distribution of the noise. The measurement noise intensity of the creep data in this study was estimated to be more than $1.4\times {10}^{-4}$ from the resolution of the linear gauge (see Appendix B). Since the influences of temperature and humidity fluctuations are added to this noise, the lower bound of the noise-intensity estimate is exist around the region where the model selection results are switched (Fig. 5(a)). This result shows the importance of specifying the measurement noise intensity in advance, even if the measurement noise intensity is only estimated as a range.

Our analysis revealed that for the Kimura model the posterior distributions of all parameters are multimodal with a small-noise-intensity prior (Figs. 6 and 7). The multimodality should result in unstable optimization when the parameters are estimated by the least-squares method, in which the parameters are optimized at a zero-noise limit. The posterior distribution of ${c}_{2}$ with a small-noise-intensity prior has a particularly multimodal and broadened distribution (Fig. 6(a4)). In fact, according to the work of Kimura *et al*.^1,2^, the estimation of ${c}_{2}$ is particularly unstable, as we explained in the previous section. Considering the present result, it would be very difficult to stably estimate not only ${c}_{2}$ but also the other parameters by the least-squares method. By contrast, for the modified Kimura model, the posterior distributions of all the parameters were unimodal regardless of the noise-intensity prior. The modified Kimura model was simplified by omitting only one parameter ${b}_{2}$. The posterior distribution of ${b}_{2}$ with small-noise-intensity prior (Fig. 7(a2)) shows that ${b}_{2}$ has a value greater than 1. This suggests that the secondary term of the Kimura model contributes to the tertiary creep as well as the secondary creep. Inevitably, the Kimura model represents the tertiary creep by three basis functions. Perhaps this is the reason for the multi modality of the posterior distribution of the Kimura model with the small-noise-intensity prior. This demonstrates that the present simplification is efficient for modifying the Kimura model toward a well-determined one. The obtained unimodality of the posterior distributions should allow us to stably estimate the parameters even by the least-squares method. Furthermore, the stability of the parameter estimation would yield a better regression of the parameters in terms of the test temperature and applied stress. Consequently, we consider that the modified Kimura model has the potential to improve the estimation of creep curves under a wide range of test conditions. These results are due to the ability of the proposed framework to capture the complex relationships among the parameters. The previous frameworks for creep, such as the framework using the Laplace approximation^13,17^, could not capture such complex relationships among the parameters.

From the posterior distributions of two parameters, we can also obtain other information about the model properties. The posterior distributions of the parameter of the exponent part and the coefficient parameter are correlated distributions (Figs. 10(a2,b2)). Mathematically, if the parameter of the exponent part increases or decreases, the coefficient parameter must decrease or increase, respectively, to maintain the same trend of the basis function. Such an analysis is useful for quantitative analysis to understand the property of the creep model.

**Figure 11:**
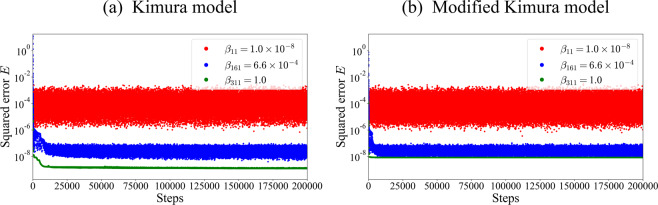
Convergence of REMC sampling. (**a**) Sampling series of squared error $E({{\boldsymbol{\theta }}}_{k},\sigma )$ of the Kimura model. Red dots represent the sampling series at low *β*_*l*_, blue is intermediate, and green is large. (**b**) Sampling series of squared error $E({{\boldsymbol{\theta }}}_{k},\sigma )$ of the modified Kimura model. Red dots represent the sampling series at low *β*_*l*_, blue is intermediate, and green is large.

Lastly, we discuss the applicability of the proposed framework to other types of creep constituent equation. The framework is computationally expensive, especially for sampling by the REMC method. In the proposed framework, the samples should be sufficiently representative such that the approximation using Eq. (26), i.e., reusing the samples for estimating the different $f({M}_{k},{\sigma }_{s})$, would be accurate. The REMC method, which is one of the generalized-ensemble algorithms, allows us to efficiently perform the marginalization of the multi modal probabilistic distribution compared with the other sampling methods^23^. We have now conservatively sampled more than 200,000 steps, but we can probably reduce it to 50,000, as shown in Fig. 11 of Appendix C. Nevertheless, applying the proposed framework to a more complex model than the Kimura model will be difficult in terms of sampling convergence. Fortunately, as we mentioned in Introduction, the Kimura model is one of the most complex creep constitutive equations^15,16^. Thus, one can apply the proposed framework to a wide range of creep constitutive equations that have been proposed so far.

Another issue is how this method applies to the case where the constituent equations are given by simultaneous differential equations. Such types of constitutive equation have been proposed on the basis of damage mechanics, such as the Hayhurst model^24^, K–R model^25^, L–M model^26^, and W–T model^27^. In principle, the proposed method is applicable to these models because the strain ${\boldsymbol{\varepsilon }}$ can be computed for a given time $t$. However, it is necessary to run a numerical simulation in each sampling step to calculate the squared error $E({{\boldsymbol{\theta }}}_{k},\sigma )$ (Eq. (11)), which takes a long computational time.

In this paper, we proposed a universal Bayesian creep model selection framework that can evaluate any type of creep constitutive equation. We also showed the effectiveness of our proposed framework using the measurement data of Gr.91 steel, a high-Cr, ferritic, heat-resistant steel. Through this evaluation, we found that the modified Kimura model could be a candidate model to improve the estimation stability of creep curves compared with the Kimura model. This was achieved by accurate estimation of the posterior distribution obtained only using the proposed framework. By applying the proposed method to different steel types at various temperatures and stresses, in-depth knowledge could be obtained about the creep deformation process and how to improve its prediction.

## Appendix A: Laplace approximation of Bayesian free energy

The log marginal likelihood function is defined as

39 $$\begin{array}{rcl}g({{\boldsymbol{\theta }}}_{k},\sigma ) & = & -\frac{1}{N}\,\log \,{\rm{P}}({\boldsymbol{\varepsilon }}|{M}_{k})\\  & = & -\frac{1}{N}\,\log [{\rm{P}}({\boldsymbol{\varepsilon }}|{{\boldsymbol{\theta }}}_{k},\sigma ,{M}_{k}){\rm{P}}({{\boldsymbol{\theta }}}_{k}|{M}_{k}){\rm{P}}(\sigma |{M}_{k})].\end{array}$$

If the Hessian matrix of the log marginal likelihood function about ${{\boldsymbol{\theta }}}_{k}$ and $\sigma$, defined as ${{\bf{H}}}^{ij}={\sum }_{i=1}^{d+1}\,{\sum }_{j=1}^{d+1}\,{\frac{{\partial }^{2}g({\Theta }_{k})}{\partial {\Theta }_{k}^{i}\partial {\Theta }_{k}^{j}}|}_{{\Theta }_{k}={\hat{\Theta }}_{k}}$, does not degenerate, the Taylor expansion around the MAP solution $({\hat{{\boldsymbol{\theta }}}}_{k},\hat{\sigma }):\,=\mathop{{\rm{\arg }}\,{\rm{\max }}}\limits_{{{\boldsymbol{\theta }}}_{k},\sigma }\,g({{\boldsymbol{\theta }}}_{k},\sigma )$ becomes

40 $$g({\Theta }_{k})=g({\hat{\Theta }}_{k})+\frac{1}{2}\,\mathop{\sum }\limits_{i=1}^{d}\,\mathop{\sum }\limits_{j=1}^{d}\,{\frac{{\partial }^{2}g({\Theta }_{k})}{\partial {\Theta }_{k}^{i}\partial {\Theta }_{k}^{j}}|}_{{\Theta }_{k}={\hat{\Theta }}_{k}}({\Theta }_{k}^{i}-{\hat{\Theta }}_{k}^{i})({\Theta }_{k}^{j}-{\hat{\Theta }}_{k}^{j})+{\mathscr{O}}({\Theta }^{3}),$$

where ${\Theta }_{k}:\,=({{\boldsymbol{\theta }}}_{k},\sigma )$ and we use the fact that the first term of the Taylor expansion becomes 0 from the definition of $({\hat{{\boldsymbol{\theta }}}}_{k},\hat{\sigma })$:

41 $$\begin{array}{l}{\frac{\partial g({\Theta }_{k})}{\partial {\Theta }_{k}^{i}}|}_{{\Theta }_{k}={\hat{\Theta }}_{k}}=0\,(i=1-d+1).\end{array}$$

By using this Taylor expansion, the saddle point approximation of $F({M}_{k})$ around the MAP solution is obtained as

42 $$F({M}_{k})=-\,\log [{\int }_{-\infty }^{\infty }d{{\boldsymbol{\theta }}}_{k}d\sigma {\rm{P}}({\boldsymbol{\varepsilon }}|{{\boldsymbol{\theta }}}_{k},\sigma ,{M}_{k}){\rm{P}}({{\boldsymbol{\theta }}}_{k}|{M}_{k}){\rm{P}}(\sigma |{M}_{k})]$$

43 $$=-\,\log \{{\int }_{-\infty }^{\infty }\,d{{\boldsymbol{\theta }}}_{k}d\sigma \,\exp [-Ng({{\boldsymbol{\theta }}}_{k},\sigma )]\}\,$$

44 $$\,\sim -\,\log \left\{\exp [-Ng({\hat{\Theta }}_{k})]\,{\int }_{-\infty }^{\infty }\,d{\Theta }_{k}\,\exp \left[-\frac{N}{2}\,\mathop{\sum }\limits_{i=1}^{d+1}\,\mathop{\sum }\limits_{j=1}^{d+1}\,({\Theta }_{k}^{i}-{\hat{\Theta }}_{k}^{i}){{\bf{H}}}^{ij}({\Theta }_{k}^{j}-{\hat{\Theta }}_{k}^{j})\right]\right\}$$

45 $$\begin{array}{rcl} & = & -\log \,{\rm{P}}({\boldsymbol{\varepsilon }}|{\hat{{\boldsymbol{\theta }}}}_{k},\hat{\sigma },{M}_{k}){\rm{P}}({\hat{{\boldsymbol{\theta }}}}_{k}|{M}_{k}){\rm{P}}(\hat{\sigma }|{M}_{k})\\  &  & -\,\frac{d+1}{2}\,\log (2\pi )+\frac{d+1}{2}\,\log (N)+\frac{1}{2}\,\log [{\rm{\det }}({\bf{H}})],\end{array}$$

where *H* is the Hessian matrix. This approximation is called the Laplace approximation. Furthermore, if we take terms whose order is higher than $logn$, Eq. (45) becomes

46 $$F({M}_{k})\sim -\,\log \,{\rm{P}}({\boldsymbol{\varepsilon }}|{\hat{{\boldsymbol{\theta }}}}_{k},\hat{\sigma },{M}_{k})+\frac{d+1}{2}\,\log (N)$$

47 $$=NE({{\boldsymbol{\theta }}}_{k},\sigma )+\frac{d+1}{2}\,\log (N),\,$$

where we use the fact that $\log \,{\rm{P}}({\boldsymbol{\varepsilon }}|{{\boldsymbol{\theta }}}_{k},\sigma ,{M}_{k})={\sum }_{i=1}^{N}\,\log \,{\rm{P}}({{\boldsymbol{\varepsilon }}}_{i}|{{\boldsymbol{\theta }}}_{k},\sigma ,{M}_{k})\equiv {\mathscr{O}}(N)$. If there is no effect on the log-likelihood $g({{\boldsymbol{\theta }}}_{k},\sigma )$ of the prior distribution, such as in the limit of $n\to \infty$, then $({\hat{{\boldsymbol{\theta }}}}_{k},\hat{\sigma })$ matches the maximum-likelihood estimator. Equation (47) is referred to as the Bayesian information criterion (BIC).

## Appendix B: Estimation of measurement error

By using the parameters of the strain gauge, i.e., the initial length ${l}_{0}$ and the length ${l}_{t}$ at time $t$, the strain ${\boldsymbol{\varepsilon }}$ is expressed as,

48 $${\boldsymbol{\varepsilon }}=\frac{{l}_{t}-{l}_{0}}{{l}_{0}}(\,=\,\hat{{\boldsymbol{\varepsilon }}}\pm \delta {\boldsymbol{\varepsilon }}),$$

49 $${l}_{0}={\hat{l}}_{0}\pm \delta {l}_{0},\,{l}_{t}={\hat{l}}_{t}\pm \delta {l}_{t},$$

where $\hat{{\boldsymbol{\varepsilon }}}$, ${\hat{l}}_{0}$ and ${\hat{l}}_{t}$ are the true values and $\delta {\boldsymbol{\varepsilon }}$, $\delta {l}_{0}$, and $\delta {l}_{t}$ are the measurement errors of each value. Because we assume that creep measurement noise $\delta {\boldsymbol{\varepsilon }}$ is added to measurement data independently and identically, the measurement error $\delta {\boldsymbol{\varepsilon }}$ propagates from $\delta {l}_{t}$ and $\delta {l}_{0}$ as follows.

50 $$\delta {\boldsymbol{\varepsilon }}=|\frac{\partial {\boldsymbol{\varepsilon }}}{\partial {l}_{0}}|\delta {l}_{0}+|\frac{\partial {\boldsymbol{\varepsilon }}}{\partial {l}_{t}}|\delta {l}_{t}=|\frac{\,-\,{l}_{t}}{{l}_{0}^{2}}|\delta {l}_{0}+|\frac{1}{{l}_{0}}|\delta {l}_{t}$$

In the measurement data used in this study, ${\hat{l}}_{0}=3\times {10}^{-2}\,[{\rm{m}}]$. From the measurement accuracy of the linear gauge of the measurement system, we find that $2\times {10}^{-6}\,[{\rm{m}}]\le \delta {l}_{0},\delta {l}_{t}$, no matter how small the estimate. Consequently, the lower limit of the measurement error of ${\boldsymbol{\varepsilon }}$ is obtained as

51 $$\delta {\boldsymbol{\varepsilon }}\ge \frac{{l}_{t}+{l}_{0}}{{l}_{0}^{2}}\times 2\times {10}^{-6}=(2+{\boldsymbol{\varepsilon }})\times \frac{2\times {10}^{-6}}{{l}_{0}}$$

52 $$\,\sim 2\times 7\times {10}^{-5}=1.4\times {10}^{-4},$$

where we assume ${\boldsymbol{\varepsilon }}\ll 2$.

## Appendix C: Convergence of REMC sampling

In this study, the samples should be sufficiently representative such that the approximation using Eq. (26), i.e., reusing the samples for estimating the different $f({M}_{k},{\sigma }_{s})$, would be accurate. Therefore, it is important to confirm the convergence of sampling at all ${\beta }_{l}$ states. We checked their sampling series at all ${\beta }_{l}$ in Figs. 11(a,b). In this study, accuracy rather than computational cost was emphasized, and conservatively long sampling, 200,000 steps, was performed. However, the sampling series of the squared error $E({{\boldsymbol{\theta }}}_{k},\sigma )$ (Eq. (11)) described in Fig. 11 shows that the sampling converges at about 50,000 steps (Figs. 11(a,b)).

## Data Availability

The data that support the findings of this study are available from Dr. M. Demura but restrictions apply to the availability of the data used under license for the current study, which are not publicly available. Data are, however, available from the authors upon reasonable request and with the permission of Dr. M. Demura.
